# Supplementary material for: Vaginal Atopobium is Associated with Spontaneous Abortion in the First Trimester: a Prospective Cohort Study in China
Source: Microbiol Spectr. 2022 Mar 21;10(2):e02039-21. doi: 10.1128/spectrum.02039-21 (PMC9045190; doi:10.1128/spectrum.02039-21)
Supplement: SUPPLEMENTAL FILE 1 — Supplemental material. Download SPECTRUM02039-21_Supp_1_seq8.pdf, PDF file, 0.5 MB [file spectrum02039-21_supp_1_seq8.pdf]

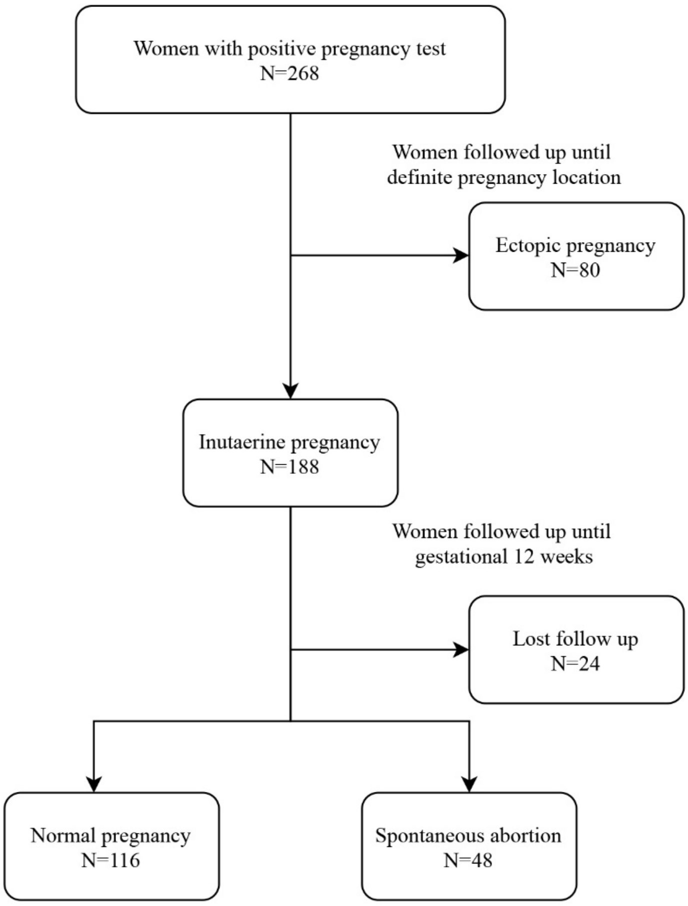

Figure S1. The flowchart of study

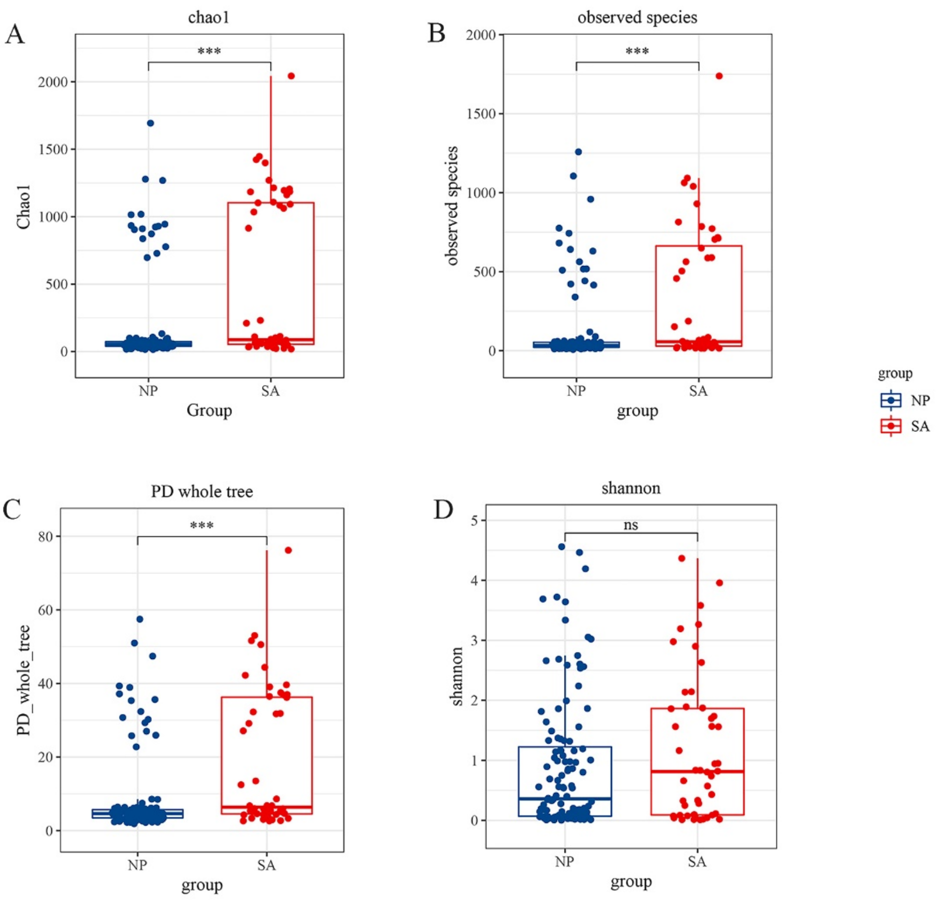

Figure S2. Alpha diversity of NP and SA group. A, Alpha diversity measured by Chao1 index. B, Alpha diversity measured by Observed species index. C, Alpha diversity measured by PD whole tree index. D, Alpha diversity measured by shannon index. Box with inside line represented interquartile range (IQR) and median, whiskers represented values within 1.5 × IQR of the first and third quartiles, points represented individual subjects. P values was calculated by Wilcoxon test.

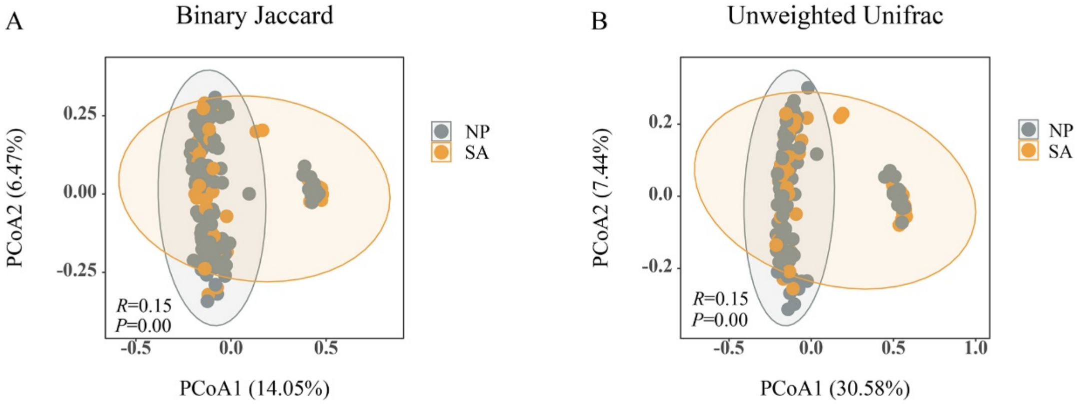

Figure S3. Beta diversity of NP and SA group. A, Principal coordinate analysis (PCoA) based on Binary Jaccard between the subjects with NP and with SA.

B, PCoA based on Unweighted Unifrac between the subjects with NP and with SA. Points represented individual subjects, and ellipses represented 95% confidence intervals around the cluster centroid. ANOSIM calculated R and P to determine the significance of clustering. Grey represented NP, orange represented SA.

Table S1 Cut point of relative abundance of *Atopobium* calculated by xgboost

Test	SA	NP	ROC area(AUC)	95%CI low	95%CI up	Best threshold	Specificity	Sensitivity
Atopobium	48	116	0.6430	0.5538	0.7321	0.0001	0.5776	0.7708
